# Decreased Insulin Sensitivity and Impaired Fibrinolytic Activity in Type 2 Diabetes Patients and Nondiabetics with Ischemic Stroke

**DOI:** 10.1155/2015/934791

**Published:** 2015-05-18

**Authors:** Aleksandra Jotic, Tanja Milicic, Nadezda Covickovic Sternic, Vladimir S. Kostic, Katarina Lalic, Veljko Jeremic, Milija Mijajlovic, Ljiljana Lukic, Natasa Rajkovic, Milorad Civcic, Marija Macesic, Jelena P. Seferovic, Jelena Stanarcic, Sandra Aleksic, Nebojsa M. Lalic

**Affiliations:** ^1^Clinic for Endocrinology, Diabetes and Metabolic Disorders, Clinical Centre of Serbia, Faculty of Medicine, University of Belgrade, Dr Subotica 13, 11000 Belgrade, Serbia; ^2^Clinic for Neurology, Clinical Centre of Serbia, Faculty of Medicine, University of Belgrade, Dr Subotica 6, 11000 Belgrade, Serbia; ^3^Department for Operations Research and Statistics, Faculty of Organizational Sciences, University of Belgrade, Jove Ilica 154, 11 000 Belgrade, Serbia

## Abstract

We analyzed (a) insulin sensitivity (IS), (b) plasma insulin (PI), and (c) plasminogen activator inhibitor-1 (PAI-1) in type 2 diabetes (T2D) patients with (group A) and without (group B) atherothrombotic ischemic stroke (ATIS), nondiabetics with ATIS (group C), and healthy controls (group D). IS was determined by minimal model (Si). Si was lower in A versus B (1.18 ± 0.67 versus 2.82 ± 0.61 min−1/mU/L × 104; *P* < 0.001) and in C versus D (3.18 ± 0.93 versus 6.13 ± 1.69 min−1/mU/L × 104; *P* < 0.001). PI and PAI-1 were higher in A versus B (PI: 19.61 ± 4.08 versus 14.91 ± 1.66 mU/L; *P* < 0.001, PAI-1: 7.75 ± 1.04 versus 4.57 ± 0.72 mU/L; *P* < 0.001) and in C versus D (PI: 15.14 ± 2.20 versus 7.58 ± 2.05 mU/L; *P* < 0.001, PAI-1: 4.78 ± 0.98 versus 3.49 ± 1.04 mU/L; *P* < 0.001). Si correlated with PAI-1 in T2D patients and nondiabetics, albeit stronger in T2D. Binary logistic regression identified insulin, PAI-1, and Si as independent predictors for ATIS in T2D patients and nondiabetics. The results imply that insulin resistance and fasting hyperinsulinemia might exert their atherogenic impact through the impaired fibrinolysis.

## 1. Introduction

Impaired insulin sensitivity (IS) plays a crucial role in the development of type 2 diabetes (T2D) [1], but its relevance for the occurrence of ischemic stroke still remains unclear. In that context, some studies suggested that decreased IS, for example, insulin resistance, is an established risk factor for ischemic stroke, [2–4] while there are studies which could not demonstrate this relationship [5].

Moreover, it has been reported that decreased IS is directly related to different subtypes of ischemic stroke in T2D patients and nondiabetics, measured by different metabolic tests, short insulin tolerance test, homeostasis model assessment for insulin resistance, and immunoreactive insulin after glucose loading in 2 h OGTT [2, 3]. Simultaneously, it has been shown that higher levels of insulin resistance were found in nondiabetics with coexistence of intra- and extracranial atherosclerosis in contrast to those with only intra- or extracranial atherosclerosis [6].

Also, it has been suggested that hyperinsulinemia might be a risk factor for ischemic stroke [7, 8]. Hyperinsulinemia represents a surrogate measure for insulin resistance in nondiabetics, as well as in T2D patients with significant residual insulin secretion capacity [9].

Simultaneously, higher levels of PAI-1 have been found in blood from patients with T2D, in obese subjects [10], and in other conditions associated with insulin resistance [11–13]. Moreover, it has been shown that hypofibrinolysis due to higher PAI-1 levels has been related to the insulin resistance [14–17] and might be involved together with insulin resistance, in the pathogenesis of ischemic stroke [18].

Therefore, in this study we tried to determine the role of impaired IS, together with the relevant changes in insulin and PAI-1 levels in T2D patients as well as nondiabetics with ischemic stroke.

## 2. Materials and Methods

### 2.1. Patients

We divided 62 T2D patients into two groups, with (*N* = 33) and without (*N* = 29) atherothrombotic ischemic stroke (ATIS), and 64 nondiabetics were assigned into group with ATIS (*N* = 34) and healthy subjects (*N* = 30), paired with the T2D patients with respect to sex and age. The diagnosis of T2D was based on the World Health Organization criteria [19]. Diagnosis of ATIS was done by a neurologist according to clinical signs and visualization methods, cranial computerized scan, and/or magnetic resonance imaging of the brain, repeated after the first 7 days from initial findings of ischemic stroke [20]. Only the patients with ATIS were included in the study, while the exclusion criteria involved patients with previously documented lacunar, cardioembolic, hemorrhagic cerebral infarction or coronary heart disease (history of myocardial infarction or coronary angiography). T2D patients were treated only with oral antihyperglycemic agents, while we excluded patients treated with insulin therapy or with other endocrine diseases, renal or hepatic insufficiency, previous and current infections, hematological or rheumatic diseases, uncontrolled hypertension, or severe alcohol consumption, during the last 4 weeks. At the time of metabolic evaluation, all the patients, irrespective of occurrence of the stroke, showed a uniform level of their physical activity.

The patients were completely informed about the study, before they gave an informed consent to participate.

The study was done at the Clinic for Endocrinology, Diabetes and Metabolic Diseases and at the Clinic for Neurology, Clinical Centre of Serbia, Faculty of Medicine, University of Belgrade, and was approved by the Institutional Ethics Committee.

### 2.2. Study Design

The obtaining of the medical history and physical examination, metabolic tests, and fibrinolytic activity evaluation were conducted in all the patients included the study within one-day visit.

Body mass index was calculated as weight in kilograms divided by the square of height in meters.

Arterial blood pressure was measured by sphygmomanometry and hypertension was diagnosed according to World Health Organisation criteria (systolic/diastolic blood pressure ≥140/≥90 mm Hg) or by the use of antihypertensive agents.

The metabolic test was performed after 6 months from occurrence of ATIS and following overnight fasting. IS was tested by using IVGTT with frequently sampled plasma glucose (PG) and PI levels, followed by the minimal model analysis [21]. The subjects were injected with glucose 0.3 g/kg body weight and the blood samples were taken immediately before intravenous glucose loading and sequentially every minute during the first 10 minutes and then 12, 14, 16, 20, 23, 24, 25, 27, 30, 40, 50, 60, 70, 80, 90, 100, 120, 160, and 180 minutes after intravenous glucose loading. Insulin was added during the test intravenously (4 mU/kg/min), between minutes 20 and 25, in order to substitute potentially diminished insulin response. The insulin sensitivity index (Si) was obtained from the data of PG and PI levels by computerized minimal model analysis (MINMOD program, kindly provided by Dr. Richard Bergman from the University of Southern California, Los Angeles).

### 2.3. Laboratory Analyses

PG was obtained by glucose oxidase method using a Beckman Glucose Analyser (Beckman Instruments). PI was measured by radioimmunoassay (INEP-Zemun) double antibody kits. Plasma PAI-1 activity was evaluated by plasminogen chromogenic plasmin substrate assay (Boehringer).

### 2.4. Statistical Analyses

Data are presented as means ± SD. Data were tested for normal distribution using Kolmogorov-Smirnov test. The continuous variables within each group of patients were analyzed with analysis of variance (ANOVA) with post hoc Tamhane test. Binary logistical regression analysis was performed. Correlation was estimated by Pearson's (*r*) correlation coefficient. The significance of the differences between correlation coefficients was analyzed by using Fisher *r*-to-*z* transformation. The differences were considered to be statistically significant at *P* < 0.05. Data were analyzed using the Statistical Package for the Social Sciences (SPSS) software (Advanced Statistics, version 17.0), Chicago, IL.

## 3. Results

### 3.1. Clinical Characteristics

The clinical characteristics of study subject are shown in Table 1. All three investigated groups of patients were matched according to the age, duration of T2D, and period of time from the onset of ischemic stroke. Simultaneously, all patients were overweight and T2D patients had satisfactory metabolic control expressed as almost equal HbA_1_c values, before metabolic investigation. The hypertension in patients with the stroke was significantly higher than in healthy controls, both in T2D patients and in nondiabetics. We did not find a significant difference in BMI between T2D patients and ATIS compared to T2D without ATIS and nondiabetics with ATIS compared to healthy subjects (Table 1).

### 3.2. Insulin Sensitivity

**Figure 1:**
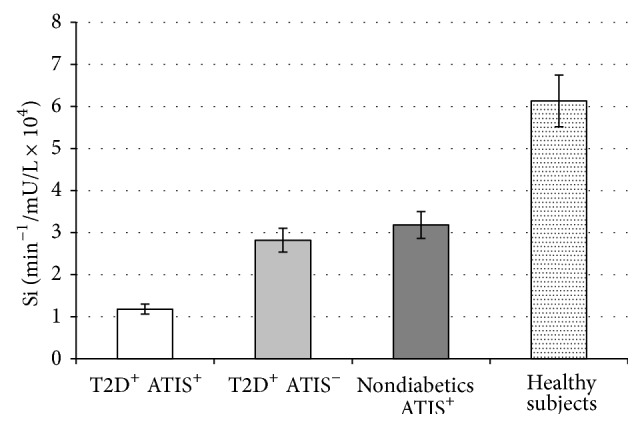
Values are means ± SE. Bar graphs show the values of Si determined by minimal model analysis. Si levels were significantly lower in type 2 diabetes (T2D) patients with atherothrombotic ischemic stroke (ATIS) compared to T2D patients without ATIS (*P* < 0.001) and in nondiabetics with ATIS compared to healthy subjects (*P* < 0.001). There is no difference in Si levels between T2D patients without ATIS and nondiabetics with ATIS (*P* = NS) (analysis of variance (ANOVA) with post hoc Tamhane test).

When we analysed IS, expressed as Si index, the lowest Si values were present in patients with T2D and ATIS and they were significantly lower in T2D patients with ATIS compared to T2D patients without ATIS (1.18 ± 0.67 versus 2.82 ± 0.61 min^−1^/mU/L × 10^4^; *P* < 0.001). Moreover, Si values were significantly lower in nondiabetics with ATIS in comparison to healthy subjects (3.18 ± 0.93 versus 6.13 ± 1.69 min^−1^/mU/L × 10^4^; *P* < 0.001) (Figure 1). There is no difference in Si between T2D patients without ATIS and nondiabetics with ATIS (*P* = NS).

### 3.3. Insulin Levels

**Figure 2:**
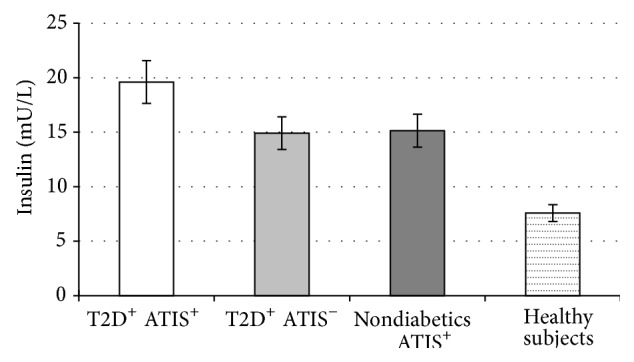
Values are means ± SE. Bar graphs show the values of basal PI level. PI levels were higher in type 2 diabetes (T2D) patients with atherothrombotic ischemic stroke (ATIS) compared to T2D patients without ATIS (*P* < 0.001) and in nondiabetics with ATIS in comparison to healthy subjects (*P* < 0.001). There is no difference in PI levels between T2D patients without ATIS and nondiabetics with ATIS (*P* = NS) (analysis of variance (ANOVA) with post hoc Tamhane test).

Simultaneously, we found that PI levels were higher in T2D patients and ATIS compared to T2D patients without ATIS (19.61 ± 4.08 versus 14.91 ± 1.66 mU/L; *P* < 0.001). In addition, PI levels were higher in nondiabetics with ATIS in comparison to healthy subjects (15.14 ± 2.20 versus 7.58 ± 2.05; *P* < 0.001). Moreover, T2D patients with ATIS showed significantly higher PI levels than nondiabetics with stroke (*P* < 0.001), but we did not document difference in PI levels between T2D patients without ATIS and nondiabetics with ATIS (Figure 2).

### 3.4. Fibrinolysis

**Figure 3:**
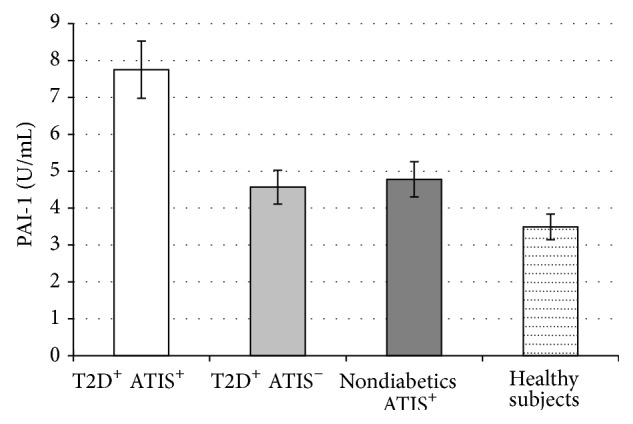
Values are means ± SE. Bar graphs show the values of PAI-1 levels. PAI-1 levels were significantly higher in type 2 diabetes (T2D) patients with atherothrombotic ischemic stroke (ATIS) compared to T2D patients without ATIS (*P* < 0.001) and in nondiabetics with ATIS compared to healthy subjects (*P* < 0.001). PAI-1 levels were not different among T2D patients without ATIS and nondiabetics with ATIS (*P* = NS) (analysis of variance (ANOVA) with post hoc Tamhane test).

Simultaneously, we found that PAI-1 levels were significantly higher in T2D patients with ATIS compared to T2D patients without ATIS (7.75 ± 1.04 versus 4.57 ± 0.72 mU/L; *P* < 0.001) and in nondiabetics with ATIS compared to healthy subjects (4.78 ± 0.98 versus 3.49 ± 1.04 mU/L; *P* < 0.001) (Figure 3). In addition, PAI-1 levels were not different among T2D patients without ATIS and nondiabetics with ATIS.

### 3.5. Correlations and Binary Logistic Regression Analysis

Moreover, we found that Si levels correlated with PAI-1 levels both in T2D (*r* = −0.690, *P* < 0.001) and in nondiabetic subjects (*r* = −0.456, *P* < 0.001) (Table 2). By using Fisher *r*-to-*z* transformation, we evaluated the difference between two correlations coefficients. The correlations of Si and PAI-1 in the T2D patients were stronger than Si and PAI-1 correlation in nondiabetics (*P* < 0.05).

The use of binary logistic regression analysis has identified levels of PAI-1, insulin, and Si as independent predictors for ATIS both in T2D patients and in nondiabetics (Tables 3 and 4).

## 4. Discussion

Our study results revealed decreased IS in patients with ATIS, whether they were T2D patients or nondiabetics, suggesting that insulin resistance may play a significant role in occurrence of the stroke. Simultaneously, T2D patients with ATIS showed the lowest level of IS, together with the fasting hyperinsulinemia. The documented hyperinsulinemia in these patients, in the settings of diminished IS levels, suggests the existence of a significant residual insulin secretion capacity. Moreover, both metabolic abnormalities, decreased IS or insulin resistance and fasting hyperinsulinemia in nondiabetics with ATIS, have confirmed the importance of decreased IS level in pathogenesis of ischemic stroke, which is consistent with the previous data [22]. Both experimental and human studies revealed the importance of insulin resistance for the occurrence of acute ischemic stroke [23–26]. However, our results signify the persistence of compromised IS and increased PI levels even 6 months after acute phase in T2D patients with ischemic stroke.

We decided to evaluate IS level in this study by using IVGTT with frequently sampled PG and PI levels with minimal model analysis, which correlates with hyperinsulinemic euglycemic clamp, previously implemented in the IS studies [27, 28].

The existence of impaired IS in different subtypes of ischemic stroke in patients with T2D has also been proposed [2]. Also, the novel study confirmed the highest values of two other parameters of insulin resistance, homeostasis model assessment for insulin resistance and insulin after glucose challenge in 2 h OGTT in patients with atherothrombotic cerebral infarction without previously documented abnormal glucose tolerance [3].

The results from extensive Atherosclerosis Risk in Communities (ARIC) Study showed that fasting insulin levels, among the other investigated risk factors, are positively associated with occurrence of ischemic stroke in the general population, which highlights the influence of insulin resistance [29], consistent with results from the Finnish cohort study that included elderly T2D patients and nondiabetics [30].

Novel data pointed out the important role of augmented insulin resistance and related metabolic abnormalities in the development of intracranial stenosis from its early stages even in nondiabetics [31].

On the other hand, lots of data suggested that PAI-1 plays a significant role in occurrence of coronary artery and cerebrovascular disease in T2D [32]. Recent studies shed new light on PAI-1 as an important pathway for cardiovascular events, including ischemic stroke [33].

Despite those facts and the background of occurrence and progression of atherosclerosis, the link between insulin resistance, diminished fibrinolytic activity, and ischemic stroke has not yet been clarified.

Our results showed higher PAI-1 levels in T2D and ATIS, in parallel with the absence of difference in PAI-1 levels between T2D patients without ATIS and nondiabetics with ATIS. However, there are some conflicting results suggesting higher PAI-1 levels in T2D without ischemic stroke compared to nondiabetics, implying the absence of further deterioration of fibrinolytic activity in ischemic stroke in T2D patients [34]. Moreover, there are indications of decreased IS, hyperinsulinemia, and increased PAI-1 in first-degree relatives of patients with ischemic stroke which is related to ethnicity, together with the findings supporting the hypothesis that diminished fibrinolytic activity may exist prior to ischemic stroke [35–37]. A previous study demonstrated that the higher PAI-1 activity in young adults with a first ischemic stroke was a consequence of acquired hypofibrinolysis [38], together with other investigations supporting the genetic control of decreased fibrinolysis [39].

Also, obese diabetics had higher PAI-1 levels compared to nondiabetics, implying permanent impaired fibrinolytic activity, which potentiates thrombosis, based on effect of combination of hormonal (hyperinsulinemia) and metabolic (hyperglycemia) changes characteristic for T2D [13, 40]. In general, increased PAI-1 level in patients with ATIS or T2D or nondiabetics could be present even 4 years after acute cerebral ischemic event [41]. Therefore, we speculate that disturbances in fibrinolysis may precede the occurrence of ATIS, having in mind higher PAI-1 level in T2D patients without ATIS and almost equal PAI-1 levels in nondiabetics with ATIS [14, 42].

We found that Si levels correlated with PAI-1 levels both in T2D and nondiabetic subjects, with stronger correlation in T2D than in nondiabetics. IS has been independently related to PAI-1 levels in our study and previously in patients with T2D and obese diabetic and nondiabetic subjects [43, 44]. In BARI 2D trial it has been shown that diminishing of insulin resistance favorably changes the balance between fibrinolysis and thrombosis potentiating fibrinolysis during long term followup in T2D patients [13]. In addition, it was suggested that patients who experience progression of symptomatic intracranial atherosclerosis are characterized by impaired endogenous fibrinolysis. Moreover, insulin resistance might be associated with recurrence of ischemic stroke [45].

PAI-1 levels may influence stroke mechanisms in multiple ways, and they might differentiate between responders and nonresponders to reperfusion therapies, and they might represent potential target for stroke prevention [46].

In order to minimize the previously confirmed harmful effect of “glucose toxicity” to the IS level and fibrinolytic activity [13, 47], we selected T2D patients with or without ischemic stroke matched with respect to duration of disease, showing optimal metabolic control before the evaluation of insulin sensitivity level. Previously described association of obesity, insulin resistance, and higher PAI-1 levels, hypersecreted from adipocytes, was the reason to include overweight subjects in our investigation [48]. Since age is known to be strongly and independently correlated with the occurrence of ischemic stroke, investigated patients were younger than 65 years. Measurements of IS were not made until at least 6 months after the stroke, providing enough time for the patients to approach maximum recovery, showing similar level of the physical activity. In order to diminish the heterogeneity of stroke, patients were matched according to the duration of ischemic brain disease.

The binary logistic regression analysis applied to our data has demonstrated that insulin, PAI-1, and Si levels are independent predictors of the ischemic stroke occurrence, in T2D patients as well as in nondiabetics.

## 5. Conclusions

In conclusion, the results demonstrated that decreased IS levels together with fasting hyperinsulinemia are strongly associated with the onset of the ATIS, while this atherogenic effect might be strongly potentiated by increased level of PAI-1. In this context, insulin resistance and impaired fibrinolytic activity might be important targets for secondary stroke prevention.

## Figures and Tables

**Table 1:** Clinical characteristics of type 2 diabetes (T2D) patients and nondiabetics with and without atherothrombotic ischemic stroke (ATIS).

	T2D^+^	T2D^+^	Nondiabetics	Healthy
	ATIS^+^	ATIS^−^	ATIS^+^	subjects
	A	B	C	D
*n* (M/F)	33 (15/18)	29 (15/14)	34 (17/17)	30 (14/16)
Age (years)	56.97 ± 2.17	58.28 ± 2.43	57.76 ± 2.75	57.46 ± 2.19
Duration of diabetes (years)	4.81 ± 1.81	5.77 ± 2.44	—	—
Period of time from onset of ischaemic stroke (years)	1.14 ± 0.39	—	1.03 ± 0.23	—
HbA1c (%)	7.36 ± 0.24	7.22 ± 0.24	5.76 ± 0.57	4.97 ± 0.45^*^
Hypertension	21 (63.6%)	18 (62.1%)	20 (58.8%)	0 (0%)^*^
BMI (kg/m^2^)	27.62 ± 3.14	27.62 ± 3.77	26.20 ± 4.09	26.39 ± 2.41

Data are *n*, means ± SD.

^∗^ *P* < 0.001, A versus B; C versus D; A versus C, D.

**Table 2:** Correlation between sensitivity index (Si) and plasminogen activator inhibitor-1 (PAI-1) levels in patients with type 2 diabetes (T2D) and nondiabetics.

T2D	*r*	*P*	Nondiabetics	*r*	*P*

PAI-1			PAI-1		

Si	−0.690	0.001	Si	−0.456	0.0001

**Table 3:** Independent factors related to T2D patients for development of ATIS in binary logistic regression analysis.

	OR (95%)	*P*
PAI-1	3.797–129.180	0.01
Insulin	3.203–5.848	0.01
Si	5.025–71.428	0.01

**Table 4:** Independent factors related to nondiabetics for development of ATIS in binary logistic regression analysis.

	OR (95%)	*P*
PAI-1	2.300–12.809	0.01
Insulin	1.443–25.526	0.014
Si	2.652–19.608	0.01
